# Prelimbic and infralimbic cortical inactivations attenuate contextually driven discriminative responding for reward

**DOI:** 10.1038/s41598-019-40532-7

**Published:** 2019-03-08

**Authors:** Sadia Riaz, Pugaliya Puveendrakumaran, Dinat Khan, Sharon Yoon, Laurie Hamel, Rutsuko Ito

**Affiliations:** 1 Department of Psychology (Scarborough), University of Toronto, Toronto, Canada; 2 Department of Cell and Systems Biology, University of Toronto, Toronto, Canada

## Abstract

The infralimbic (IL) and prelimbic (PL) cortices of the medial prefrontal cortex (mPFC) have been shown to differentially control context-dependent behavior, with the PL implicated in the expression of contextually conditioned fear and drug-seeking, and the IL in the suppression of these behaviors. However, the roles of these subregions in contextually driven natural reward-seeking remain relatively underexplored. The present study further examined the functional dichotomy within the mPFC in the contextual control over cued reward-seeking, using a contextual biconditional discrimination (CBD) task. Rats were first trained to emit a nose poke response to the presentation of an auditory stimulus (e.g., X) for the delivery of sucrose reward, and to withhold a nose poke response to the presentation of another auditory stimulus (e.g., Y) in a context-specific manner (e.g. Context A: X+, Y−; Context B: X−, Y+). Following acquisition, rats received bilateral microinjections of GABA receptor agonists (muscimol and baclofen), or saline into the IL or PL, prior to a CBD training session and a probe test (under extinction conditions). Both IL and PL inactivation resulted in robust impairment in CBD performance, indicating that both subregions are involved in the processing of appetitively motivated contextual memories in reward-seeking.

## Introduction

The effective processing of contextual information plays a paramount role in guiding adaptive behavior that is essential to an organism’s survival. For instance, animals need to adapt their foraging and mating behavior to rapidly changing environments, and the emission of socially appropriate behaviors is highly context dependent. Aberrant contextual processing can have adverse consequences, as seen in post-traumatic stress triggered by otherwise safe contexts and context-induced drug relapse^1,2^. Despite this, the precise underlying neural circuitry subserving the processing of contextual information remains to be elucidated^3–6^.

The medial prefrontal cortex (mPFC) is a central component of the affective cortico-limbic-striatal system that is thought to regulate behavior motivated by both reward and aversive outcomes^7^. The rodent mPFC is an anatomically and functionally heterogeneous structure consisting of the prelimbic (PL) and infralimbic (IL) cortices, which have largely distinct projection patterns to the nucleus accumbens, amygdala and hypothalamus^8–10^. However, both PL and IL receive robust projections from the ventral hippocampus (HPC)^11–13^, which is strongly implicated in the processing of appetitive and aversive contextual information^14–21^. The PL and IL are therefore well placed to monitor changing environmental contexts, in order to engage in the orchestration of the most contextually-appropriate behavior through their efferent projection sites. Indeed, the mPFC has been implicated in context-induced reinstatement of drug seeking^1,22^ and the expression of contextual fear^23^. Furthermore, these studies have uncovered a functional dichotomy between the PL and IL in contextual fear processing and context-induced reinstatement of drug seeking: while activity in the PL has been shown to promote the expression of contextual fear and drug seeking behavior, IL activation has been associated with the extinction and inhibition of contextual fear and drug seeking^22–26^. However, the existence of this functional dichotomy in appetitively motivated contextual memory processing in the presence of natural rewards (i.e., in the absence of drugs of abuse) is not as well investigated, as very few existing studies have conducted differential PL and IL manipulations in the same study^27–29^.

Thus, the present study further investigated the role of the PL and IL subregions of the mPFC in context-dependent cued reward-seeking that has previously been demonstrated to be impaired by ventral, but not dorsal hippocampal lesions^21^. We investigated the effects of transient, post-acquisition GABAR_A&B_ agonist-mediated inactivation of the PL and IL on a contextual biconditional discrimination (CBD) task in which contextual information (Contexts A & B) is used to disambiguate the reinforcement contingencies of two discriminative cues. Animals were trained to emit an instrumental nose poke response to the presentation of one auditory stimulus (e.g. X+) for a sucrose reward, and to withhold responding to the presentation of a second stimulus (e.g. Y−) in a context-dependent manner (e.g. AX+, AY−; BX−, BY+). Upon successful CBD memory acquisition, animals were subjected to inactivation of the PL or IL during a CBD training session as well as a CBD probe test (in the absence of outcomes). We observed significant deficits in CBD memory retrieval following both PL and IL inactivation, which were not due to changes in locomotor activity, motivational states, or deficits in the ability to process discrete cues. Our findings help to bridge the gap in the existing literature by providing evidence for the causal role of PL and IL cortices in appetitively motivated contextual processing in the presence of natural rewards.

## Results

### Verification of cannulae placement

**Figure 1:**
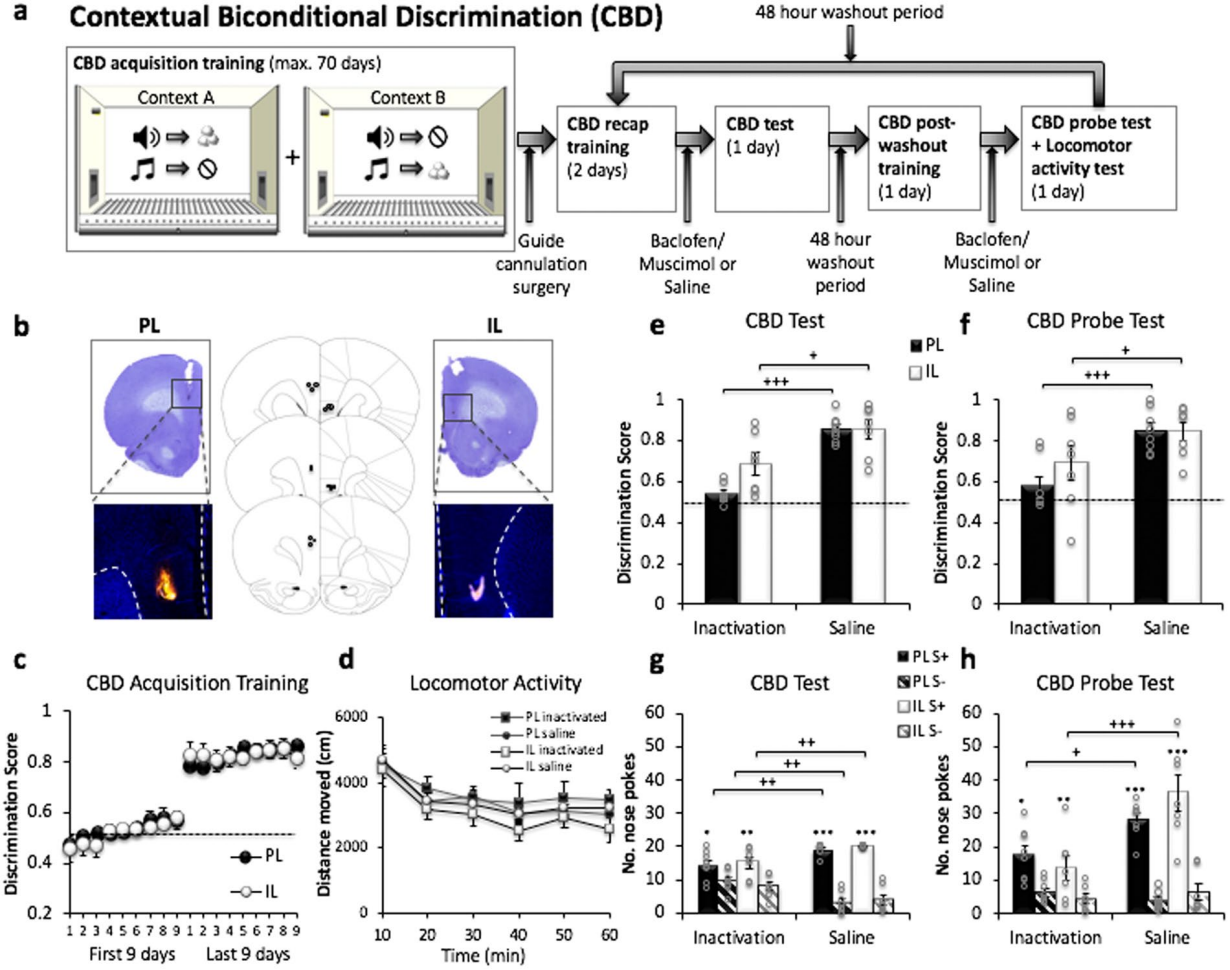
Contextual Biconditional Discrimination (CBD) task. (**a**) Overview of experimental procedure. Animals were trained to receive reward (sucrose pellets) by nose poking (>0.5 s) into a magazine inside the operant box. During CBD acquisition training, animals were trained to associate two distinct auditory cues with an appetitive outcome (sucrose) or no outcome (house light off), in a context dependent manner. After CBD memory acquisition, animals received bilateral guide cannula implantation surgery, before being subjected to saline and drug (inactivation) treatment testing cycles in a within-subjects experimental design. Each testing cycle (order counterbalanced) began with 2 days of CBD recap training. Once stable CBD memory expression was established, animals received bilateral infusions of either saline or GABAR agonists and underwent a CBD test (with outcomes). After a 48-hour washout period, animals were once again trained on the CBD task, before receiving bilateral infusions (as before) and undergoing a CBD probe test (without outcomes). Each animal underwent two pharmacological treatment cycles (saline and inactivation) separated by a 48-hour washout period. (**b**) Schematic diagrams and representative photomicrographs showing the position of the injector tip and drug spread in the PL and IL of animals used for the CBD task (PL n = 8, IL n = 7). Drug spread measured using fluorophore-conjugated muscimol had an estimated radius of 0.3 mm for a 0.3ul infusion in both IL and PL. (**c**) Animals that were later assigned to the 2 cannulation groups showed significant learning (pre-surgery) from the first 9 days to the last 9 days of CBD acquisition training (p < 0.001). (**d**) Effect of PL and IL inactivation on locomotor activity. Mean distance moved over 10 min intervals (cm) ± SEM is plotted for each treatment cycle, for both cannulation groups. (**e–h**) Mean discrimination scores (correct nose poke holds during the S+/total nose pokes during S+ and S–, out of 40 trials in total) or mean number of nose-poke hold responses emitted during S+ and S− trials (20 each), averaged across the 2 contexts ± SEM are plotted. The dotted line depicts discrimination performance at chance level. Both PL and IL inactivation significantly impaired CBD performance in the presence of outcomes (e,g, CBD test, p < 0.001) and in the CBD probe test administered under extinction conditions (f,h, CBD probe test, p < 0.001). Asterisks above bars denote significant within subject differences in performance (drug vs. saline) - *^,+^*p* < 0.05, **^,++^*p* < 0.01, ***^,+++^*p* < 0.001.

**Figure 2:**
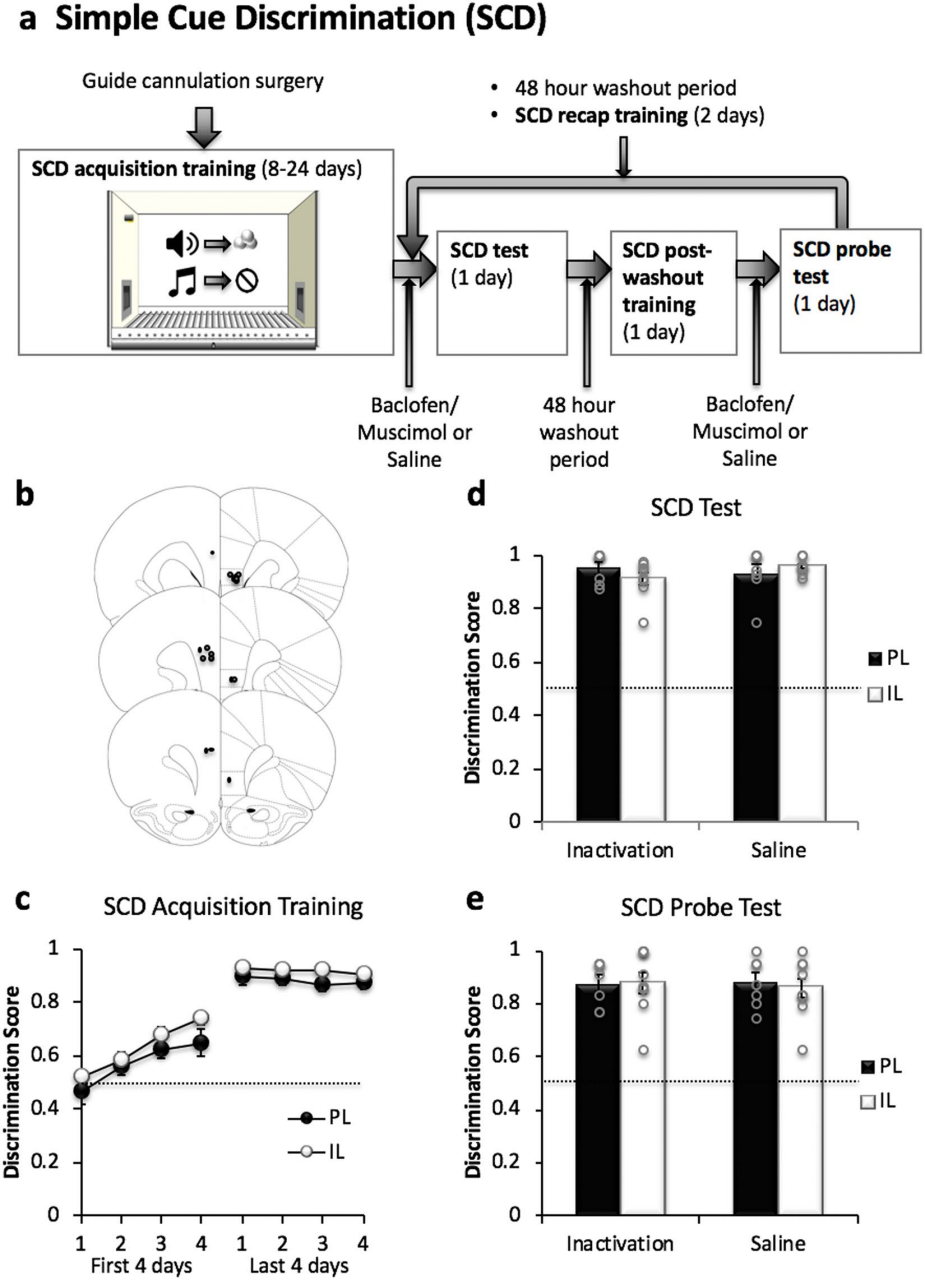
Simple Cue Discrimination (SCD) task. (**a**) Overview of the task procedures. Prior to training on the SCD task, animals received bilateral guide cannula implantation surgery. Animals received magazine and nose poke hold (0.5 s) trainings in one operant chamber whereby they were trained to receive reward by nose poking (>0.5 s) into a magazine in the operant chamber. During SCD acquisition training, animals were trained to associate two distinct auditory cues with an appetitive outcome (sucrose) or no outcome (house light off), before being subjected to saline and drug (inactivation) treatment testing cycles in a within-subjects fashion. Each testing cycle (order counterbalanced) began with bilateral infusions of either saline or GABAR agonists and SCD training. After a 48 hr washout period, animals were once again trained on the SCD task, before receiving bilateral infusions (as before) and undergoing a SCD probe test. Each animal underwent two pharmacological treatment cycles (saline and inactivation) separated by a 48-hour washout period and 2 days of SCD recap training. (**b**) Schematic diagrams showing the position of the injector tip in the PL and IL of animals used for the SCD and PR tasks (PL n = 9, IL n = 9). (**c**) Acquisition of SCD memory. All animals showed significant learning from the first 4 days to the last 4 days of CD training (p < 0.001). Both PL (n = 7) and IL (n = 9) groups reached a similar level of SCD learning by the end of the acquisition training. (**d**–**e**) Effect of PL and IL inactivation on discriminative responding in the presence of outcomes (SCD test, d; PL n = 7, IL n = 9) and under extinction conditions (SCD probe test, e; PL n = 7, IL n = 8). There was no significant effect on SCD performance (in the presence of outcomes and under extinction conditions) following inactivation of either region indicating that neither PL nor IL inactivation affected discrete cue processing in this task. Mean discrimination scores ± SEM are plotted for panels c-e.

Figures 1b and 2b show schematic diagrams^30^ and representative photomicrographs of the approximate drug spread and placement of the injector tip within the PL and IL, for animals included in the CBD task (Fig. 1b) and animals used in a simple cue discrimination (SCD) and progressive ratio reward-seeking (PR) tasks (Fig. 2b). The spread of drug (muscimol/baclofen) within the PL and IL was estimated to be 0.1 mm (radius) per 0.1ul of drug volume injected (0.3 mm radius total) using the microinfusion of 0.3ul of flurophore-conjudated muscimol, which is consistent with previous findings of drug spread measured using the same agent in the hippocampus^31^ as well as a functional assay of drug action as measured by cfos activation surrounding the injector tip^32^. Thus, the present results are unlikely to have been the outcome of the drug spreading to adjacent regions.

#### Contextual Biconditional Discrmination (CBD) Task

Magazine training and nose poke hold (0.5 s) training: All animals acquired the instrumental nose poke hold responses (≥0.5 s) in the active receptacle to obtain a sucrose reward, achieving the learning criteria (obtaining 50 rewards within the 20 min session, in both contexts) within two days of training. Furthermore, all rats nose poked preferentially into the active receptacle (Magazine; *F*(1, 13) = 27.417, *p* < 0.001), regardless of the context or the cannulation group they were later assigned to (Context (*F*(1, 13) = 1.368, *p* = 0.263, Region (that was later cannulated) (*F*(1, 13) = 0.727, *p* = 0.409), no significant interactions (*p* > 0.05)).

CBD training: Animals were then trained to acquire discriminative nose poke hold responses to two auditory cues (S+, S−) in two different contexts (A or B), with the reward contingencies of the stimuli reversed in the two contexts (Fig. 1a). Learning was assessed on the basis of a criterion performance of obtaining a discrimination ratio score of >0.75 in each context for 5 consecutive days of training. Since the number of days of CBD acquisition training ranged from 18 to 65 days, only data from the first 9 and last 9 days of training were further analyzed (Fig. 1c).

Significant learning was observed between the first 9 days and last 9 days of CBD training across both contexts (Days: *F*(17, 221) = 65.968, *p* < 0.00001). Furthermore, there were no baseline differences in CBD learning in each context prior to intra-cerebral pharmacological manipulations. (Context, *F*(1, 13) = 0.029, *p* = 0.867, Region (which was later cannulated) *F*(1, 13) = 0.075, p = 0.788, nor any significant interactions (all *p* > 0.05)).

Stable baseline CBD memory expression: We compared the CBD training data obtained at several different time points in the experiment (Fig. 1a) to ensure that CBD performance remained stable after surgical cannula implantation, and each round of mircoinfusions. Thus, the discrimination ratios from the last day of CBD training, 2 days of recap training after recovery from surgery, 2 days of post-washout training (after each treatment cycle) and CBD training following saline infusion, revealed that CBD memory retrieval was consistent across these 6 days (Days: *F*(7, 91) = 1.435, *p* = 0.201) and across the two contexts (Context: *F*(1, 13) = 0.01, *p* = 0.920), for both the PL and IL groups (Region: *F*(1, 13) = 0.113, *p* = 0.742). There were no significant interactions between any of the factors (*p* > 0.05).

CBD test performance was impaired following PL and IL inactivation: A within subject comparison of the CBD discrimination scores across two CBD training sessions, one prior to which the rats received micro-infusions of saline and another prior to which the rats received the drug muscimol and baclofen (M/B), showed that GABAR_A & B_ agonist-mediated inactivation of the PL and IL both significantly reduced the CBD discrimination scores in both contexts (Treatment; *F*(1, 13) = 39.698, *p* < 0.0001, Context; (*F*(1, 13) = 0.024, *p* = 0.880, Region; *F*(1, 13) = 4.366, *p* = 0.057, no significant interactions; all *p* > 0.05). Additional t-tests comparing the saline and inactivation treatments for each region were conducted to fully address our *a priori* research question; PL and IL inactivation both significantly impaired performance on the CBD test (*p* < 0.001 and *p* = 0.011, respectively, Fig. 1e).

Furthermore, the mean number of nose pokes made during each type of stimulus presentation across the two sessions (drug vs. saline) (Fig. 1g) was compared and it was found that the infusion of the drug M/B caused significant changes in the pattern of responding to S+ and S− in both contexts (Stimulus type x Treatment; *F*(1, 13) = 35.761, *p* < 0.0001). More specifically, while animals in both treatment groups nose poked preferentially to the S+ stimulus (Stimulus type, *F*(1, 13) = 175.858, *p* < 0.0001), saline treated animals made significantly more nose pokes during the S+ presentation (*p* < 0.001), and significantly fewer nose pokes to the S− presentation (*p* < 0.01), in comparison to the drug-treated conditions. Additional post-hoc tests comparing responses made during the S+ and S− stimuli were conducted to elucidate the nature of the impairment observed; although PL and IL inactivation both significantly impaired performance on the CBD test, all groups showed a significant preference for the S+ stimulus (PL inactivation: *p* = 0.021; IL inactivation: *p* = 0.003; PL saline: *p* < 0.001; IL saline: *p* < 0.001, Fig. 1g). PL inactivation resulted in a significant decrease in responding to the S+ (*p* = 0.004) and increase in responding to the S− stimulus (*p* = 0.005), in comparison to the PL saline treatment condition, whereas IL inactivation resulted in a significant decrease in responding to the S+ (*p* = 0.006), but no significant change in responding to the S− (*p* = 0.078), in comparison to the IL saline treatment condition.

CBD probe test performance was impaired following PL and IL inactivation: A CBD probe test was also conducted to assess CBD performance under extinction conditions (cue presentations alone), across two sessions in which the animals received saline or drug microinfusions into the PL or IL prior to each. Inactivation of the PL and IL caused a significant attenuation of cue discrimination in both contexts (Treatment; *F*(1, 13) = 33.162, *p* < 0.0001, Context; *F*(1, 13) = 4.537, *p* = 0.053, Region; *F*(1, 13) = 0.681, *p* = 0.424, no significant interactions; all *p* > 0.05). In order to fully address our *a priori* research question, additional t-tests comparing the saline and inactivation treatments for each region were conducted; PL and IL inactivation both significantly impaired performance on the CBD probe test (*p* < 0.001 and *p* = 0.014, respectively, Fig. 1f).

The number of nose pokes made during each type of stimulus presentation (Fig. 1h) in the two sessions was also significantly different following drug microinfusions in the IL, but not the PL group (Treatment; *F*(1, 13) = 24.123, *p* < 0.001, Treatment x Stimulus type; (*F*(1, 13) = 30.387, *p* < 0.001, Treatment x Region; (*F*(1, 13) = 5.915, *p* = 0.03). While all animals emitted more responses to S+ than S− (Stimulus type (*F*(1, 13) = 67.392, *p* < 0.001), IL drug infusions caused animals to emit less nose pokes than IL saline infusions overall (*p* < 0.001). There was no significant difference in the overall levels of nose poking between the PL inactivated and saline-infused groups (*p* = 0.093). Post-hoc tests comparing responses made during the S+ and S− stimuli were conducted to elucidate the nature of the observed impairment; although PL and IL inactivation both significantly impaired performance on the CBD probe test, all groups showed a significant preference for the S+ stimulus (PL inactivation: *p* = 0.003; IL inactivation: *p* = 0.015; PL saline: *p* < 0.001; IL saline: *p* < 0.001, Fig. 1h). Both PL and IL inactivation resulted in a significant decrease in responding to the S+ (*p* = 0.020 and *p* < 0.001, respectively) compared to saline treatment, without a significant change in responding to the S− (*p* = 0.189 and *p* = 0.251, respectively).

#### Locomotor Activity was unaffected following PL and IL inactivation

Drug infusions into the PL and IL did not induce significant alterations in spontaneous locomotor activity across the 4 treatment groups (PL inactivated, PL saline, IL inactivated, IL saline; Fig. 1d, Time; *F*(5, 65) = 23.491, *p* < 0.00001, Treatment; *F*(1, 13) = 0.171, *p* = 0.686, Region; *F*(1, 13) = 1.035, *p* = 0.328, no significant interactions; all *p* > 0.05).

#### Simple Cue Discrimination (SCD) Task

A separate cohort of animals was trained to acquire discriminative cue responding in one context only in order to investigate the possibility that the PL and IL inactivation effect observed in contextual biconditional discrimination reflects an impairment in simple discriminative cue responding (Fig. 2a). All rats acquired the nose poke hold responses (≥0.5 s), showing significant preference for nose poking into the active magazine (*F*(1, 14) = 145.245, *p* < 0.00001) within two days of training. D**i**scriminative cue responding was assessed based on criteria learning (ratio score of > 0.75 for 5 consecutive days) and the number of days of acquisition training ranged from 8 to 24 days. Thus, only data from the first 4 and last 4 days of training were analyzed (Fig. 2c).

A comparison of the discrimination scores from the first 4 and last 4 days of SCD acquisition training revealed significant learning taking place across the training days in all groups of animals (*F*(7, 98) = 67.494, *p* < 0.00001, Days x Region: *F*(7, 98) = 0.305, *p* = 0.950). Baseline levels of SCD performance was reestablished between the two treatment cycles (saline vs drug, Days: *F*(5, 70) = 1.310, *p* = 0.270, Region: *F*(1, 14) = 0.087, *p* = 0.773, Days*Region: *F*(5, 70) = 0.753, *p* = 0.587; overall ANOVA compared the last day of SCD acquisition training, 2 days of CD retraining, 2 days of post-washout training (both treatment cycles) and SCD training following saline infusions).

Performance on the SCD test and SCD probe test was unaffected following PL and IL inactivation: Figure 2 shows the data from the SCD test (with outcomes, Fig. 2d) and the SCD probe test (without outcomes, Fig. 2e). Neither PL nor IL inactivation had any effect on the discrimination scores from the SCD test (Treatment; *F*(1, 14) = 0.078, *p* = 0.784, Region; *F*(1, 14) = 0.038, *p* = 0.848, Treatment x Region: *F*(1, 14) = 0.118, *p* = 0.737). These data were further supported by the data from the SCD probe test, which showed no effect of PL or IL inactivation on discrimination scores during the probe test (Treatment: *F*(1, 13) = 0.319, *p* = 0.582, Region: *F*(1, 13) = 0.122, *p* = 0.733, Treatment*Region: *F*(1, 13) = 2.001, *p* = 0.181).

#### Progressive Ratio (PR) Task

**Figure 3:**
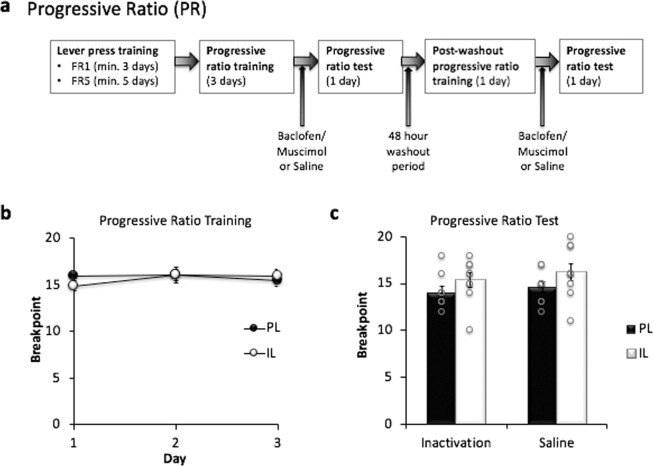
Progressive Ratio (PR) task. (**a**) Overview of the task procedures. Following magazine and lever press training, animals underwent 3 days of PR training before receiving bilateral infusions of either saline or GABAR agonists and PR training (Progressive Ratio Test). After a 48 hr washout period, animals were once again trained on the PR task, before receiving bilateral infusions of GABAR agonists or saline (respectively) and a final PR training session (PL n = 9, IL n = 9). (**b**) All rats demonstrated stable performance during the Progressive Ratio acquisition training. Mean breakpoints ± SEM are plotted in panels b-c for both cannulation groups when subjected to each treatment condition (drug and saline) in a within-subjects experimental design. (**c)** Effect of PL and IL inactivation on progressive ratio (PR) performance. There was no significant effect on PR performance following inactivation of either region indicating that neither PL nor IL inactivation has any effect on motivational states.

Rats were trained to lever press for sucrose pellets on a progressive ratio schedule of reinforcement to assess the effects of PL and IL inactivation upon the motivation to lever press for reward (Fig. 3a). There was no difference in the baseline PR performance of rats prior to drug/saline infusion days (last three days of training, Days: *F*(2, 32) = 1.374, *p* = 0.268, Region: *F*(1, 16) = 0.055, *p* = 0.818, Days x Region: *F*(2, 32) = 1.417, *p* = 0.257, Fig. 3b).

Another ANOVA comparing breakpoints from the last day of PR training, post-washout PR training and PR training following saline infusion revealed that baseline performance on the PR task was stable across these 3 days in both the PL and IL groups (Days: *F*(2, 32) = 2.537, *p* = 0.095, Region: *F*(1, 16) = 1.824, *p* = 0.196, Days*Region: *F*(2, 32) = 1.220, *p* = 0.309). These data also establish that performance on the PR task did not deviate from baseline performance following saline infusions in both cannulation groups.

PR test performance was unaffected following PL and IL inactivation: Figure 3c shows breakpoint data from the PR test. Inactivation of the PL and IL had no effect on PR performance, in comparison to the saline treatment (Treatment: *F*(1, 16) = 2.005, *p* = 0.171, Region: *F*(1, 16) = 2.279, *p* = 0.151, Treatment x Region: *F*(1, 16) = 0.109, *p* = 0.745).

## Discussion

The present study examined the role of the PL and IL subregions of the medial prefrontal cortex (mPFC) in context-dependent biconditional discrimination (CBD) performance, using post-training reversible inactivation in rats undergoing CBD under non-extinction and extinction conditions. It was found that both PL and IL are critically involved in the contextual retrieval of reward cue memory. Inactivation of either region significantly impaired responding for the reward-associated discriminative stimulus (S+) in both contexts during the probe test (under extinction conditions). Furthermore, the presence of the outcomes (reward or timeout) did not diminish this impairment, indicating that the PL and IL are also important for the expression of context-guided discriminative cue responding. Importantly, GABAR_A&B_ mediated inactivation of the PL and IL had no significant effect on discriminative cue responding *per se*, the motivation to respond for natural reward or locomotor activity. Thus, our findings indicate that the PL and IL are both engaged in the use of contextual information in adaptive responding for natural rewards and reward-associated cues.

### Both PL and IL cortices are important for context-appropriate discriminative cue responding

The present study implicates both subregions of the mPFC, PL and IL in the contextual control of appetitively cued instrumental responding. A closer examination of the nature of the observed deficit in discriminative responding revealed that PL and IL inactivation caused significantly diminished context-specific nose-poke responding to the S+ (reward cue) compared to the level of responding following saline injections, under conditions in which the reward outcome accompanied responding and under extinction conditions. In contrast, context-specific responding during the presentation of the S− *increased* after PL inactivation, but only when responding with the outcomes present. The level of responding to the S− was not significantly impacted after IL inactivation both under extinction and non-extinction (reward present) conditions, albeit the difference in S− responding following inactivation and saline infusions approached significance (p = 0.078) under the latter condition. Thus, overall, the degree of impairment in context-specific discriminative cue responding was more pronounced when animals received PL or IL inactivation while responding for rewarded outcomes. The involvement of the PL in higher order cue control of instrumental responses has been previously reported, with muscimol-induced PL inactivation leading to impairment in context-specific suppression of responding for reward pellets in the presence of cues that signal shock versus non-shock in a contextual biconditional discrimination task using fear stimuli, as opposed to reward-associated cues^25^. Similarly, the PL has been implicated in the use of contextual cues to resolve response conflict in a dual biconditional discrimination task^26^.

However, the contribution of the IL in the contextual control of instrumental responses is not as well documented. In fact, the present results are somewhat at odds with the popular dichotomous view of PL/IL function that the PL is involved in the expression/promotion of context- or cue-elicited reward or fear-related bahaviors, and the IL in the suppression of these bahaviors after extinction learning. However, it is important to note that this widely supported view is based predominantly on the findings of studies observing context-induced reinstatement of drug seeking^1,22,33^ and the expression of contextual or cued fear^23,34^. Delineating the inherent role of PL and IL in context processing in animals having undergone prolonged exposure to drugs of abuse can be tenuous, given the widely reported drug-induced changes in brain function and neuronal changes^35,36^. Furthermore, studies implicating the PL and IL in opposing roles in conditioned fear have utilized tasks that are Pavlovian, wherein a conditioned stimulus, after being associated with an aversive outcome such as a foot-shock, will elicit a startle or freezing response. Findings from studies that have examined the effects of manipulations in the PL/IL on the expression of more complex operant responses such as active avoidance are more equivocal regarding their putative functional dichotomy. For instance, an experiment by Bravo-Rivera *et al*. (2014) found that inactivation of the PL resulted in an attenuation of the expression of active avoidance – the emission of an escape bahavior in response to a conditioned stimulus associated with a foot-shock, while leaving freezing behavior intact^37^. In contrast, IL inactivation was found to disrupt the extinction of avoidance bahavior. Conversely, in a study by Moscarello and LeDoux (2013), IL inactivation decreased active avoidance and increased conditioned freezing even in the absence of extinction history^38^. These findings suggest that under more complex experimental conditions, both the PL and IL may be involved in the expression of conditioned fear behavior, warranting further research into their roles in motivated behavior under the control of operant or Pavlovian contingencies.

Only a handful of existing studies have specifically investigated the role of the PL and IL in contextual processing during natural reward seeking^28,29^. Ashwell and Ito^29^ trained rats with PL or IL excitotoxic lesions on a contextual discriminative responding task, whereby responding to a discrete light cue in 3 out of 6 radial maze arms (spatial contexts) was rewarded with sucrose delivery, while responding to the cue in the other 3 locations was not rewarded. It was found that IL-lesioned animals showed facilitated acquisition of the context-dependent discriminative cue approach bahavior and facilitated reversal learning (reversal of context-dependent reward contingency) in comparison to the PL lesioned group, which was impaired in the acquisition of context retrieval of appetitive cue memory. The divergence of the present findings from these results could be explained by a number of key differences across the two studies such as the examination of acquisition vs. expression of context-dependent instrumental responding and a difference in the operational definition of the ‘context’, with the use of proximal cues, as opposed to spatial cues in the present study. Previous pharmacological, genetic and biochemical data indicate that acquisition and retrieval processes can recruit different molecular mechanisms and neural circuits^39–41^. The CBD task is also markedly more complex as its performance requires the use of configural associations^4,42,43^.

Similarly, our IL inactivation data differ from the findings of Villaruel *et al*.^28^ who demonstrated that unilateral optogenetic stimulation of the IL during the presentation of a Pavlovian cue previously associated with sucrose (in the same context, A) and then extinguished in a separate context (B) reduced context-induced renewal of conditioned responses to the Pavlovian cue. In this case extinction was achieved prior to a test of contextual memory renewal, which is different from testing retrieval of an acquired contextual memory without extinction (as in the CBD task). Even within extinction learning paradigms, the neural mechanisms recruited during context-dependent cue memory renewal may differ based on the context in which the renewal occurs (i.e., in the same or different context than the one in which extinction occurred^44^, and the temporal delay between memory acquisition and extinction^45^. Additionally, as discussed earlier, the observed differences could be attributed to differential recruitment of the IL in instrumental (present task) and Pavlovian (Villaruel *et al*.) tasks.

Nevertheless, the pattern of results from the present study is supported by neuroanatomical evidence that both the PL and IL subregions of the mPFC receive robust projections from the ventral hippocampus (vHPC)^11–13^, but not the dorsal hippocampus^46–48^. We have previously shown the vHPC but not the dorsal HPC, is necessary for appetitively motivated contextual processing using the same CBD task^21^, and propose that contextual information is conveyed through the projections from the ventral HPC to the PL and IL to enable animals to respond appropriately to changing reward contingencies (discriminative responding). Recent work has also implicated the ventral HPC CA1 to mPFC (PL and IL) pathway in higher order regulation of food intake and reward-seeking behavior mediated by glucagon 1 peptide signaling in the ventral HPC, substantiating the notion that the PL and IL work cooperatively along with the ventral HPC in mediating adaptive control over natural reward-seeking^49^. Additionally, Moorman and Aston-Jones (2015) reported that neuronal activity in both PL and IL is correlated with ‘contextually-appropriate’ reward-seeking during rewarded trials, and the withholding of lever pressing during non-rewarded (extinction) conditions^50^. Together with further evidence that the PL and IL show highly interactive and synchronized fast oscillatory activity that disappears when the two subregions are disconnected^51^, the present finding of PL and IL involvement in context-specific discriminative cue responding under conditions of extinction and outcomes present, suggest that both the IL and PL operate as a functional unit in monitoring contextually relevant/changing contingencies and orchestrating the most appropriate behavioral response.

### PL and IL cortices are not necessary for discriminative cue responding

It is unlikely that the observed deficits in CBD performance were due to an impairment in the PL-and IL-inactivated animals to discriminate between a sucrose-associated S+ and non-reward S−, as we found no evidence of an impairment in simple (context-independent) discriminative cue responding following PL and IL inactivation. These findings are consistent with previous studies showing that lesions of the mPFC do not impair simple discrimination learning^52,53^. Similarly, amongst studies that have specifically targeted the PL or IL, Sangha *et al*.^54^ showed intact preferential nose-poke responses to a Pavlovian sucrose-cue in comparison to the presentation of fear- or safety-cues following PL and IL inactivation with microinjections of muscimol/baclofen. Other studies examining the role of mPFC in appetitive cue processing have found that drug-associated cue processing relies on the mPFC^55–58^. However, as alluded to earlier, exposure to drugs of abuse likely induces pathological alterations to the way that mPFC may be engaged in the processing of drug-associated cues or context, and therefore does not allow us to draw conclusions about the inherent roles of the PL and IL in reward-seeking under drug-free conditions.

We have also demonstrated that the observed effects of PL and IL inactivation on the contextual retrieval of natural reward cue memory and performance of contextual biconditional discrimination are not mediated by changes in the motivation to respond for natural reward, or in locomotion. Sparing of motivation for natural rewards following mPFC inactivation has been demonstrated previously in a study by Ball and Slane^59^ who reported that inactivation of PL or IL did not affect food self-administration bahavior under a FR5 schedule. Our locomotor activity test results are also in agreement with existing literature reporting no significant changes in locomotion following inactivation of the PL or IL^60–62^.

In conclusion, the present study provides strong evidence for a role for PL and IL in exerting contextual control over discriminative cue responding for natural reward, which is highly congruent with the widely held view of the mPFC being important in the optimization and allocation of resources to adapt to rapidly changing environments^63,64^. Despite the prevailing view of the PL and IL subserving dissociative, and sometimes even opposing functions in the expression of conditioned reward-seeking and conditioned fear, the present findings suggest that the PL and IL work cooperatively under circumstances in which contextual information conveyed from the vHPC is required to modulate responding to behaviorally relevant environmental cues.

## Methods

### Subjects

36 experimentally naïve, male, adult Long-Evans rats (Charles-River Laboratory, Canada) were used in this study. All rats were maintained at 90% of their free-feeding weights for the duration of the experiment (350–450 g) and had access to water *ad libitum*. Animals were pair-housed in a room held at a constant temperature of 22 °C and relative humidity of 30–60%, under a 12 h light/dark cycle. All experiments were conducted during the light phase, between 0700 and 1900 h, in accordance with the guidelines of the Canadian Council of Animal Care, and were approved by the University of Toronto Local Animal Care Committee.

### Apparatus

Six operant boxes (Med Associates, Georgia, VT), housed in sound-attenuating and light-resistant chambers were used in this experiment. Each operant box had a floor made of stainless steel rods (0.5 cm diameter rods, spaced 1.6 cm apart), and two sidewalls containing a recessed food magazine in the center, one of which was associated with the delivery of 45 mg sucrose pellets (i.e., the active [right] receptacle, TestDiet, Richmond, IN). Each food magazine was equipped with an infrared beam detector to monitor the number, timing and duration of nose pokes made into the magazine. In addition, a 2 kHz Sonalert tone generator was mounted high on the wall opposite the wall with the active receptacle. A white noise generator was also affixed lower down on the same wall. The chamber was illuminated by a house light (28 V) mounted on the top left wall (center).

The six boxes were divided into two sets of three boxes to represent two different ‘contexts’ based on a number of distinguishing features; the dimension and appearance of the chambers [Med Associates chambers ENV01: Set 1: 30 cm (W) × 20 cm (H) × 20 cm (D) vs. Med Associates chambers ENV08: Set 2: 30 cm (W) × 20 cm (H) × 25 cm (D)] and the odors of the chambers (Set 1: Sandalwood, Set 2: Bitter Almond). The respective odors were present within each box during all training and test sessions. Each operant box was cleaned with an odorless 1% Liquinox solution (Alconox, White Plains, NY) before and after each session to remove any traces of sucrose or odors from the previous rat in the same box.

All operant boxes were controlled via a computer with MED-PC software (Med Associates), which also automatically recorded the data generated during the experiment.

### Behavioral procedures

#### Contextual Biconditional Discrimination (CBD) Task

18 animals were trained in the contextual biconditional discrimination (CBD) task (see Fig. 1 for overview of procedures), as described previously^14^.

Habituation: All rats received two 20 min sessions in which they were exposed to one of each type of operant box (1 and 2). Context assignments were carefully counterbalanced for box type; for 9 rats the small/sandalwood chambers (Set 1) served as context A and the large/bitter almond chambers (Set 2) served as context B, while the context assignment was reversed for the remaining 9 rats. During habituation and for each subsequent training day, the order of context presentation was changed across days (e.g., A-B, B-A, B-A, A-B). After the habituation sessions, all rats were exposed to three sucrose pellets (per rat) that were placed in their home cage to overcome any neophobia.

Magazine training: Following habituation, all rats received one session of magazine training in each context to learn to retrieve sucrose pellets from the active receptacle. Each session lasted for 20 min during which a total of 60 sucrose pellets were delivered on a variable interval 20-second schedule (VI20). The number of nose pokes made into each receptacle (active or inactive) was recorded.

Nose poke hold training: Each rat received a maximum of 2 days (four sessions; one session per context per day) of nose poke hold training. During each session, successful nose pokes (held for ≥ 0.5 s) in the active receptacle were rewarded on a continuous reinforcement (Fixed Ratio 1) schedule. An inter-response interval (latent period) of 10 s followed each successful nose poke during which no rewards were dispensed. Nose poke holds in the inactive receptacle had no consequence. Each session lasted for 20 min or until a maximum of 50 sucrose pellets were dispensed. Once a subject obtained all 50 rewards within the 20 min session, in both contexts, they were transferred to the next phase of behavioral training.

CBD training: Rats received a maximum of 70 days of CBD training, in which they were trained to acquire discriminative nose poke hold responses in two different contexts. In one context (e.g., A), the tone served as the reinforced discriminative auditory stimulus (S+) and the white noise as the non-reinforced discriminative auditory stimulus (S–) while in the other context (e.g., B) the contingencies were reversed. Each rat received two 25–30 min sessions of training each day (one per context). Each session consisted of a total of 40 trials (20S+ and 20S–), and began with a 90 s pre-stimulus period. Each trial began with the presentation of the S+ or S– for a maximum of 7.5 s. A nose poke hold (for ≥ 0.5 s) emitted in the active receptacle during an S+ presentation resulted in the delivery of three sucrose pellets, followed by the termination of the auditory stimulus 1 s later. In contrast, a nose poke hold for ≥ 0.5 s in response to the S– resulted in a 5 s timeout period with the house light off and the session timer paused. Nose poke holds in the inactive receptacle had no consequence. In the absence of any successful responses, the auditory stimuli terminated after 7.5 s. The inter-trial interval (ITI) was set at 30 s. The order of S+ and S– presentation was pseudo-randomized to ensure that the same stimulus was not presented for more than two consecutive trials in each session (e.g., S+, S–, S–, S+, S–, S+, S+, S–…). The number of nose pokes made during the presentation of each type of stimulus was recorded. In order to control for any baseline differences in locomotor activity, a discrimination score was used to assess CBD memory acquisition. The discrimination score was calculated for each rat, per day, by dividing the number of successful responses during the S+ by the total number of nose poke holds emitted during the S+ and S– in each context and averaging the ratio scores from the two contexts.

All animals underwent CBD training until they obtained a ratio score of > 0.75 in each context for 5 consecutive days of training, within a maximum of 70 days of training.

Guide cannula implantation surgery: All rats underwent bilateral cannula implantation after acquiring the contextual biconditional discrimination. Each rat was anesthetized with isoflurane gaseous anesthetic (3% isoflurane delivered in O_2_ at 1 L min^−1^; Baxter, Mississauga, ON). The rat was then placed in a stereotaxic frame (Kopf Instruments, Tujunga, CA) with the incisor bar set at 3.3 mm below the interaural line. A small scalp incision was made to implant guide cannulae (26 gauge; Plastics One, Roanoke, VA) bilaterally into the prelimbic cortex (in mm from Bregma: AP +2.2, ML ±0.75, DV −2.5; PL group, n = 10) or the infralimbic cortex (in mm from Bregma: AP +2.2, ML ±0.75, DV −3.4; IL group, n = 8), according to Paxinos and Watson^26^. The cannulae were secured on the skull using dental cement (Lang Dental, Wheeling, IL) and 3 anchoring screws (Plastics One, Roanoke, VA). In order to maintain the patency of the guide cannulae, solid stainless steel dummy cannulae (Plastics One) were inserted into the guide cannulae following surgery. All rats were given a 7-day post-operative recovery period before continuing CBD training.

In a within-subjects experimental design, 15 (of 18) animals underwent the remaining bahavioral procedures twice- once with a saline treatment and once with a drug (inactivation) treatment. The order in which rats received the two cycles of testing was counterbalanced across animals, e.g., 4 rats received the PL inactivated treatment cycle first, followed by a 48 hr washout period and then the PL saline treatment cycle, while this order was reversed for the remaining PL animals. 1 rat from each cannulation group was excluded from the study due to loss of headcap prior to the completion of the inactivation treatment cycle. In addition, 1 rat from the PL cannulation group was excluded due to cannula misplacement.

CBD (recap) training: Training was resumed after the postoperative recovery period for a total of 2 days for CBD recap training. All rats underwent the same training procedure as before to ensure that the surgery and post-operative rest period did not affect the expression of CBD memory. In the second cycle of testing, animals underwent recap training to ensure that the extinction test (see below) did not affect CBD memory expression.

General infusion procedure: On the second day of recap training, each rat was infused with 0.3 ul saline solution per side (0.9% saline; B. Braun Medical, Bethlehem, PA) to minimize the mechanical effects of subsequent drug infusions, as well as to habituate the animal to the infusion procedure. All infusions were made at a rate of 0.3 ul/min using an infusion pump (Harvard Apparatus, Holliston, MA) mounted with a 5 ul Hamilton syringe. The injector tip used (33 gauge; Plastics One) for PL and IL infusions projected 1 mm and 1.5 mm (respectively) below the tip of the guide cannula. Following each injection, the needle was left in place for 1 min to allow for diffusion of the drug or saline away from the injector tip and to minimize its spread along the needle tract. For all subsequent infusions, each rat was given a 15 min interval before the start of behavioral testing to allow the drug to take effect.

CBD test: Targeted brain regions were temporarily inactivated using drug MB, a gamma-aminobutyric acid A and B (GABA_A_ and GABA_B_) receptor agonist cocktail of muscimol and baclofen (in equal parts at a concentration of 250 ng/ul), respectively. In the inactivation treatment cycle, rats were infused with 0.3 μl (75 ng) of MB per side, while in the saline treatment cycle, rats were infused with 0.3 μl of saline solution per side. Following infusions, rats received two sessions of CBD training, one per context, as described above.

CBD postwashout training: Following the test day, rats received a two-day washout period (48 hr) before an additional retraining session per context was administered to all rats to ensure that the infusions did not have a lasting effect on the expression of CBD memory.

CBD probe test: The following day, rats were infused with MB or saline solution again (as above) before testing the effect of PL or IL manipulation on the expression of CBD memory in the absence of outcomes. The operant boxes and test stimuli used in the probe test were identical to those used in the CBD training sessions, except for a change in the total number of trials administered (20; 10S+ and 10 S–) and the duration of stimulus presentation (10 s). There was no consequence to nose poking to stimuli presented during the probe test. Each rat received one session of the probe test per context. Half of the rats in each treatment group (PL saline, PL inactivated, IL saline and IL inactivated) were tested in context A and then context B and this order was reversed for the remaining rats. Successful (held for ≥ 0.5 s) and unsuccessful nose pokes were recorded separately although neither had any consequence during the presentation of either auditory stimulus. As before, only responses held for ≥ 0.5 s were used to calculate the discrimination scores.

#### Locomotor activity test

Following the second probe test session, all rats were administered a locomotor activity test in opaque plastic chambers measuring 45 cm × 25 cm × 20 cm. A video camera and EthoVision XT software (Noldus, Wageningen, The Netherlands) were used to measure the total distance travelled by each rat (in cm) over a 60 min period. Distance traveled was recorded in 10-minute bins. Locomotor activity measurement of the saline groups was used as a baseline for comparison with the locomotor activity of each corresponding inactivated group (drug-infused).

#### Simple Cue Discrimination Task

18 experimentally naïve animals were used for a simple discriminative responding task (SCD) in which rats were trained to acquire discriminative nose poke hold responses in the presence of two discriminative stimuli (tone and white noise), in order to assess whether PL or IL manipulation affects discriminative cue processing, without the need for contextual discrimination. The SCD task was adapted from the CBD task described above such that each animal was trained in a single context only (Fig. 2). Half the animals in each cannulation group were trained in operant chamber set 1, while the other half were trained in set 2.

Animals received PL (n = 9) and IL (n = 9) guide cannulation surgery before habituation, magazine training and nose poke hold (0.5 s) training. Rats underwent SCD acquisition training until they obtained a discrimination score of > 0.75 for 5 consecutive days of training, within a maximum of 40 days of training. Once this learning criterion was reached, animals received infusions of MB or saline before a SCD test (with outcomes), a 48 hr washout period and a SCD post-washout training session. The same infusion was administered again before a final SCD probe test to complete the treatment cycle. Following the infusion, animals had a 2-day washout period and 2 days of SCD recap training, before the second treatment cycle (infusion and SCD test, 48 hr washout, SCD post-washout training, and infusion and SCD probe test). Two rats from the PL group were excluded from the SCD task due to failure to meet the SCD acquisition learning criteria. SCD probe test data from one IL cannulated rat was excluded as the animal became non-responsive in both probe tests.

#### Progressive Ratio (PR) Task

After the CD task, rats were given a 2-day washout period before being run on a progressive ratio (PR) task to assess the effects of PL/IL manipulations on the motivation to respond for reward (Fig. 3). The PR task utilized Med Associates operant chambers with a retractable lever on either side of the right (active) receptacle.

Magazine training: Rats received one session of magazine training to learn to retrieve sucrose pellets from the active receptacle. The session lasted for 20 min during which a total of 60 sucrose pellets were delivered on a VI20 schedule.

Lever press training: Rats were trained to lever press on a fixed ratio (FR) 1 reinforcement schedule, whereby each lever press resulted in the delivery of one sucrose pellet. A lever was extended at the start of the session (the side of the lever was counterbalanced across animals) and retracted for a 6-second ITI each time a response was made. The session ended after 30 minutes or when 50 sucrose pellets had been delivered. Rats where transferred to the next phase of training once they obtained 50 pellets within 30 min, with a minimum of 3 FR1 training sessions.

Following FR1 training, rats underwent a minimum of 5 days of FR5 training, which lasted for 45 min or until 50 pellets were delivered. ITI was maintained at 6 sec. Rats were transferred to the progressive ratio training after 5 sessions, if they received 50 pellets within the 45 min session.

PR training: Rats were trained on a PR schedule of reinforcement, whereby the number of lever presses required to attain a sucrose pellet increased systematically according to the formula *[5e*^(R*0.*2)*^*] − 5* (rounded to the nearest integer), where *R* = *number of sucrose pellets already delivered* + *1*^65^. The number of lever presses required to receive a sucrose pellet reward were 1, 2, 4, 6, 9, 12, 15, 20… etc. There was a 5 sec ITI following each reward delivery where the lever was retracted and no responses could be emitted. As before, the side of the lever was counterbalanced across animals. The PR training session ended after 1 hour or when the animal withheld responding for 10 min. The final ratio completed during the session was measured as the breakpoint. Each animal underwent 3 days of PR training to establish baseline performance.

PR test: The effect of PL or IL manipulation on motivation was measured using a within subjects experimental design whereby rats received infusions of 0.3ul of either MB or saline before a PR training session, followed by a 48 hr washout period, a post-washout PR training session, and infusions of either saline or MB (respectively) before a final PR training session.

### Histology

Following the completion of behavioral testing, rats were then given a lethal dose of Euthanyl (2 mL/4.5 kg; Bimeda, Cambridge, ON) and were intracardially perfused with 100 ml saline, followed by 100 ml of 4% paraformaldehyde solution (PFA; Sigma-Aldrich) to fix the brain. Brains were then removed, stored in PFA, and transferred to a 30% sucrose cryoprotectant solution before sectioning. All brains were cut coronally in 50 um slices, and stained with cresyl violet for the verification of cannula and injector tip placements via comparison with the rat brain atlas of Paxinos and Watson^26^.

For verification of drug spread, 2 experimentally naïve animals with bilateral PL and IL cannulation (respectively) were infused with 0.3ul of fluorophore-conjugated muscimol 15 min before perfusion. These brains were stored in phosphate buffered saline, sliced coronally (50 um slices) and coverslipped with Fluoroshield Mounting medium with DAPI for the visualization of the fluorophore under a fluorescent microscope (visualized with a TRITC filter).

### Data analysis

SPSS statistical package version 20.0 (SPSS, Chicago, IL) was used for all statistical analyses with the level of significance set at p < 0.05. Mixed design analysis of variance (ANOVA) was carried out on data collected from each phase of the experiment, with the region (PL, IL) as the between-subjects factor. The within-subjects factors varied across tasks and are described individually for each task in the Results section. Several two-tailed t-tests planned *a priori* were also conducted to examine the data generated from the PL and IL groups separately.

## Data Availability

The datasets generated during and/or analysed during the current study are available from the corresponding author on reasonable request.
